# ApoER2 trafficking, processing and signaling and its participation in neurodegeneration

**DOI:** 10.1186/1750-1326-7-S1-L11

**Published:** 2012-02-07

**Authors:** María-Paz Marzolo

**Affiliations:** 1 Depto. Biología Celular y Molecular, Fac. Ciencias Biológicas and MINREB, Pontificia Universidad Católica de Chile, Chile

## Background

ApoER2, a member of the LDL receptor family, binds to reelin, triggering a signaling pathway that regulates neuronal migration and positioning in the developing brain and dendritic ramification. In the adult, reelin participates in neuronal survival, synaptic plasticity and neurogenesis. Our interest has been to study cell biology aspects of ApoER2, including the molecular and cellular determinants of its endocytic trafficking, how these features regulate the receptor signaling properties and ApoER2 participation in neurodegenerative conditions, such as Alzheimer’s Disease and in the Niemann Pick Type C disease (NPC), a lipid storage and neurodegenerative disorder caused by genetic mutations in *npc1* gene that causes cholesterol accumulation in late endosome/lysosomes. In NPC there are alterations in the trafficking and signaling of some receptors however this remains unexplored for ApoER2. As APP, ApoER2 is also processed by proteases and these events are regulated by ligand binding and some cytosolic proteins such as Dab1. By yeast two-hybrid it was found that ApoER2 binds to SNX17, a cytosolic protein that regulates trafficking of membrane proteins in the early endosomal pathway. However SNX17 role in ApoER2 trafficking and signaling has not been evaluated.

## Methods

*Endocytic trafficking* (internalization and/or recycling) and *processing* of ApoER2 was evaluated in cell lines transfected with different forms of the receptor to determine the role of 1) its NPxY motif and proline rich insert present in the cytoplasmic domain, 2) Lipid rafts association, 3) dominant negative expression or silencing of adaptor proteins such as Dab2 and SNX17 4) Intracellular cholesterol accumulation. *ApoER2 signaling* in different conditions (silencing of SNX17; late endosome/lysosome cholesterol accumulation mimicking the NPC condition) were performed in primary cultured neurons activated by reelin, analyzing dendritic branching and determinations of signaling proteins by Western blot.

## Results

ApoER2 internalization is clathrin mediated, determined by its NPXY motif and the adaptor protein Dab2. Lipid rafts association or the presence of the proline-rich insert do not modulate the receptor internalization. The NPXY motif is also responsible of the recycling of ApoER2 from the early endosome, which is mediated by SNX17. Abolishing SNX17 affects ApoER2 signaling in neurons and the receptor processing. ApoER2 processing is stimulated by its own ligand reelin but also by activation of other signaling receptors. In cellular conditions miming NPC there was a decrease in ApoER2 surface levels, explained by a more efficient internalization and less recycling. NPC neurons, exhibited less dendritic ramification while the number of apoptotic cells was increased. Both effects were partial but significantly rescued when exogenous Reelin was added. We found relevant differences in the expression of ApoER2 and its ligand reelin in the brains of the NPC KO mice.

## Conclusions

ApoER2/Reelin signaling depends on the receptor’s trafficking and, in NPC, signaling is partially but significantly active indicating that it could act as a survival mechanism in this neurodegenerative disease. Supported by FONDECYT 1110382 and MINREB.

